# Deconstructing Job Insecurity: Do its Qualitative and Quantitative Dimensions Add Up?

**DOI:** 10.1007/s41542-021-00096-3

**Published:** 2021-08-12

**Authors:** Ieva Urbanaviciute, Jurgita Lazauskaite-Zabielske, Hans De Witte

**Affiliations:** 1 Swiss National Centre of Competence in Research LIVES, University of Lausanne, Bâtiment Géopolis, CH-1015 Lausanne, Switzerland; 2 Institute of Psychology, University of Lausanne, Bâtiment Géopolis, CH-1015 Lausanne, Switzerland; 3 Institute of Psychology, Vilnius University, Vilnius, Lithuania; 4 Research Group Work, Organizational and Personnel Psychology, Leuven, KU Belgium; 5 Optentia Research Focus Area, North-West University, Campus, Vanderbijlpark, South Africa

**Keywords:** Job insecurity, Latent profile analysis, Employee well-being, Organizational change, Psychosocial stress

## Abstract

Despite substantial interest in job insecurity as a severe workplace stressor, the way in which its qualitative and quantitative dimensions co-occur is not fully understood. As a result, the variety of their combinations and potentially differential effects that they produce remain underexplored. The current study aimed to address this gap in two ways. First, we hypothesized that quantitative job insecurity would manifest in a cumulated form along with qualitative job insecurity but not vice versa. Second, we aimed to test whether different combinations of quantitative and qualitative job insecurity differentially reflect in employees’ occupational characteristics and health and well-being outcomes.

Latent profile analyses were conducted on two different samples of employees (*N* = 1077 and *N* = 608). The findings from both samples supported a three-profile solution of qualitative and quantitative job insecurity resulting in the balanced low, balanced high, and qualitative job insecurity dominant profiles. As expected, the probability of temporary and part-time employment was the highest in the balanced high (i.e., cumulated) job insecurity profile. Moreover, both the balanced high and the qualitative job insecurity dominant profiles were linked to significantly lower mental health and well-being than the low job insecurity profile, suggesting that substantially detrimental effects may occur even if experiencing qualitative job insecurity only.

**Supplementary Information:**

The online version contains supplementary material available at 10.1007/s41542-021-00096-3.

In the turbulent world of work, job insecurity has become a major stressor with compelling evidence of its far-reaching detrimental effects on employee health and well-being (De Witte et al. 2016; Probst 2005). An increasing number of studies thus aim to understand the ways in which job insecurity manifests. In doing so, it is necessary to account for the multidimensional nature of the construct as its different dimensions may occur simultaneously and the prevalence and potentially differential effects of such configurations remain largely understudied.

To elaborate on the above, the current study relies on a psychological perspective on job insecurity, which considers it a subjective perception rather than a purely objective phenomenon. One of the most widely used psychological definitions of job insecurity distinguishes between its qualitative and quantitative dimensions (Hellgren et al. 1999). Qualitative job insecurity refers to the perceived threat of losing valued features of the job, whereas the quantitative dimension pertains to the anticipated loss of the employment situation as a whole (Greenhalgh and Rosenblatt 1984; Hellgren et al. 1999). While there is some debate about the perceptual boundaries between these two dimensions (e.g., Probst 2005), there is no doubt they can go side by side and research usually agrees that they represent different aspects of insecurity in employment relationships (Sender et al. 2017). Conceptually, the target of quantitative job insecurity is larger in scope, which means that it possibly overarches qualitative job insecurity, so that they are both present in a cumulated form. However, consistent observation of higher mean levels of qualitative than quantitative job insecurity when measured together (Chirumbolo et al. 2017; Tu et al. 2019) suggests that qualitative job insecurity may be relatively more salient and occur rather independently from quantitative job insecurity.

Unfortunately, despite some recent attempts to test an integrated model encompassing both dimensions (Chirumbolo et al. 2017), the knowledge about the ways in which they combine is scant. To date, the majority of studies have relied on a variable-centered approach, which inspects correlations between variables across individuals rather than looking into how quantitative and qualitative job insecurity may combine and be simultaneously experienced by the same person. Only recently, De Cuyper et al. (2019) have offered a rare exception. Their study has revealed the existence of five distinct combinations of job insecurity with differential effects on career outcomes, thereby showing the utility of a person-centered perspective in better understanding job insecurity as a stressor. This line of research certainly merits further elaboration because it can shed new light on the variety of job-insecure situations and their severity as well as their prevalence within the population. Linking them to employees’ characteristics that pertain to occupational instability and further inspecting the scope of their detrimental effects allows for a better detection of high-risk employee groups, which is relevant both in theory and in practice.

To this end, the present study has set a three-fold goal. First, it aimed to test whether different person-centric combinations of quantitative and qualitative job insecurity (i.e., latent profiles) identified by De Cuyper (2019) could be empirically justified and replicated. Second, it additionally hypothesized that the two insecurity dimensions might be not mutually inclusive in their manifestation (i.e., quantitative job insecurity usually also implies qualitative job insecurity, but the latter might occur on its own). Third, it sought to expand on the investigated correlates testing how different job insecurity profiles link to objective occupational characteristics and selected well-being outcomes (i.e., work engagement, exhaustion, and mental health). Objective “instability” characteristics are important to investigate because they may determine the occurrence of job insecurity (e.g., Keim et al. 2014; Shoss 2017) that is considered a labor market vulnerability (De Witte et al. 2015), whereas the above-mentioned well-being outcomes are key indicators of employee thriving versus strain and have been commonly associated with job insecurity (Probst 2002; Sverke et al. 2002; Vander Elst et al. 2014 a and b). We thus contribute to the stressor-strain literature by examining how well different job insecurity profiles (versus separate dimensions) differentiate between situations with ranging employee precarity and testing whether there is any evidence that the most extreme profiles that are high on both dimensions produce cumulative negative effects.

## Qualitative and Quantitative Job Insecurity

The distinction between qualitative and quantitative job insecurity was introduced in the early works of Greenhalgh and Rosenblatt (1984) and was further elaborated by Hellgren et al. (1999). Currently, it is one of the most widely adopted conceptualizations of job insecurity^1^ used in numerous psychological studies. However, despite the definition of job insecurity as a multidimensional phenomenon, to date, most attention has been given to its quantitative dimension that refers to the loss of the job as such (De Witte et al. 2015). Scholars have argued that due to uncertainty about the future and powerlessness to change the situation, a threat of unemployment (i.e., quantitative job insecurity) is a severe work stressor with a host of detrimental outcomes for both employees and their organizations (Probst 2002; Sverke et al. 2002). Qualitative job insecurity, on the other hand, seems to be a milder form of insecurity, as it does not imply the job loss but rather some unwanted changes in it or the loss of its valued features (Hellgren et al. 1999). Nevertheless, it is considered a particularly relevant aspect of job insecurity in today’s changing world of work (Lee et al. 2018). It has been shown to trigger a wide range of detrimental effects on employee attitudes and behaviors with similar underlying psychological mechanisms as those of quantitative job insecurity (Stynen et al. 2015; Vander Elst et al. 2014a and b). The current literature unequivocally recognizes that both job insecurity dimensions separately represent a risk for employees’ and organizational well-being (see Shoss 2017 for a recent overview). However, it rarely takes into account that, when analyzed jointly, quantitative and qualitative job insecurity define a situational scenario that can encompass either one or both dimensions to a varying degree, and thus may vary in its precarity from person to person. This is an important moment to inspect from a strain-stressor point of view. Conservation of Resources Theory (Hobfoll 2001) is often used to explain the detrimental nature of job insecurity by resource depletion that is at the core of strain experience. Both continuous employment (the subject of quantitative job insecurity) and valued features of the job (the subject of qualitative job insecurity) can be thought of as resources that employees aim to maintain and protect. Yet, they represent *separate* forms of resources, which implies that strain and precarity may add up when they are threatened altogether. Such situation requires much more effort to cope with and leaves less space to rely on other available resources to keep things in balance (e.g., in the case of qualitative job insecurity, one may still invest in the future of one’s job, but this is obviously not possible if both forms of insecurity are present). For this reason, we are in need of a better understanding of how the two aspects of job insecurity may combine into a unique personal experience and what implications it bears for occupational precarity and well-being.

Somewhat surprisingly, there is only fragmented information on how qualitative and quantitative job insecurity manifest together and what their relative salience is. Some indirect evidence comes from studies that have included both dimensions measured on the same scale. Many of them have observed slightly higher mean levels of qualitative than quantitative job insecurity (Chirumbolo et al. 2017; De Cuyper et al. 2010; De Witte et al. 2010). This offers a data-driven suggestion that situations of qualitative job insecurity may occur on their own (i.e., quantitative job insecurity can remain low even if qualitative job insecurity is high). On the other hand, vice versa is not necessarily true as the two dimensions are arguably not isolated from one another. As maintained by Greenhalgh and Rosenblatt (1984) and more recently reemphasized by De Witte (2015), quantitative job insecurity has a wider scope in terms of what is lost. Hence, in theory, whenever a person experiences quantitative job insecurity, it should come with qualitative job insecurity because the loss of employment often implies the loss of all valued features of the current job.

A recent study by Chirumbolo et al. (2017) presented one way of testing this via an integrated model where quantitative job insecurity was suggested to affect employee outcomes through qualitative job insecurity (i.e., it mediated or explained the detrimental effects of its quantitative counterpart). This is a notable finding because it marks one of the first attempts to explicitly investigate how the two job insecurity dimensions come into effect and suggests that at some point they converge. However, sequential effects only partially address the questions raised above. They demonstrate an overall tendency for qualitative job insecurity to follow quantitative job insecurity from a variable-centric perspective but do not inform about the severity of such situation for a given person. To do so, we need to know how elevated job insecurity was at the moment when its two aspects (co-)occurred. Hence, in order to truly understand job insecurity as a cumulative stressor, it may be useful to take a closer look into the different level patterns of these *co-occurrences* for they may considerably vary from person to person.

A variable-centered approach has some limitations in serving this type of inquiry. Notably, it relies on the correlations of variables on which people differ but does not capture well how they characterize different people, or how a given pattern of variables is simultaneously experienced by the same person. Moreover, pattern analyses using variable-centered methods are often based on pre-defined combinations of indicators of interest (e.g., by contrasting the focal predictor-outcome associations at selected values of an interacting variable or creating and comparing certain categories), which may be somewhat restrictive in estimating the outcomes. In this case, a person-centered approach is advantageous because it models a configuration of multiple indicators, thus “allowing researchers to understand how variables operate conjointly and within people” (Gabriel et al. 2015, p. 865).

## A Person-Centered Approach to Job Insecurity

A person-centered analytic approach represents a group of methods that share a key feature of assuming population heterogeneity. Latent profile analysis (LPA) is one such method aimed at identifying latent (i.e., directly unobserved) subpopulations or profiles within a population based on the patterns of the investigated set of theoretically related, yet distinct variables (Howard & Hoffman 2018; Zyphur 2009). While various clustering methods are certainly not new in psychology, compared to traditional clustering analyses LPA treats individual profile membership as an unobserved categorical variable, with a varying degree of probability of belonging to a given profile (Spurk et al. 2020). It is considered an ideal technique for investigating qualitatively and quantitatively different patterns that may differ in their shape (i.e., the way in which profile indicator variables are configured above or below the sample mean across the profiles) and levels (i.e., the absolute levels of the profile indicator variables; for details see Gabriel et al. 2015; Spurk et al. 2020). As such, LPA is capable of yielding important benefits because it offers more specificity than a regression-based approach drawn from a single population (Howard and Hoffman 2018), and it focuses on the nature of the individual situation rather than separate variables (Zyphur 2009). This is particularly applicable to the topic of our current study, where we see a benefit in capturing the most common, empirically justified personal job insecurity situations in terms of combinations of qualitative-quantitative job insecurity, as well as identifying those that are less likely to occur.

De Cuyper et al. (2019) have demonstrated the viability of such approach. Their study used latent profile analyses to classify their sample into several profiles based on different combinations of high to low quantitative and qualitative job insecurity. Their findings supported the idea that as much as five such profiles may exist defining distinct situations of job insecurity that range from relatively secure to very insecure. Hence, these analyses offer a completely new insight allowing to see what exactly happens when the two dimensions coincide, irrespective of their sequence. While De Cuyper et al. (2019) did not consider a broad range of occupational well-being antecedents and outcomes, their findings yielded first evidence that different career implications could be expected across the job insecurity profiles and they were substantially less favorable in more severe profiles.

We opted for a similar approach in the current study. In doing so, we first of all expected to replicate the variety of distinct latent profiles found by De Cuyper et al. (2019). We furthermore advanced on this line of reasoning by testing a hypothesis that the qualitative and quantitative dimensions may combine in a cumulative but *not mutually inclusive* way, which has not been done thus far. Given that quantitative job insecurity is presumably larger in scope in terms of loss (Greenhalgh and Rosenblatt 1984), and that it is thought to encompass (De Witte et al. 2015) or even trigger qualitative job insecurity (Chirumbolo et al. 2017), we expected to find a “cumulated” profile that would characterize employees experiencing high quantitative job insecurity *along with* high qualitative job insecurity. However, according to our hypothesis, the reverse would be not true, as by definition high qualitative job insecurity does not necessarily imply the loss of the job itself. In this way, we approach the integration of qualitative and quantitative job insecurity (cf. Chirumbolo et al. 2017) from a different point of view by focusing on *concurrent* combinations of the two dimensions as stated below:

*H1a:* A set of job insecurity profiles exist that are distinct in their levels and shape.

*H1b:* Qualitative and quantitative job insecurity combine in a cumulative but not mutually inclusive way, so that high quantitative job insecurity profile implies high qualitative job insecurity but not vice versa.

## Covariates of the Job Insecurity Profiles

### Contextual Covariates

To date, a large body of research has been dedicated to the investigation of both situational and psychological correlates of job insecurity. Understanding the context is extremely important for it provides essential information about when and for whom job insecurity is most likely to occur, thereby enabling researchers and practitioners to pinpoint the sources of job insecurity. The literature identifies a number of objective conditions that either predict or covary with job insecurity. Among others, age, tenure, contract type, occupational category, organizational communication, and organizational change experience have been associated with the likelihood of experiencing job insecurity (Keim et al. 2014; Näswall and De Witte 2003), whereas age and tenure have also been observed to moderate some of its detrimental outcomes (Cheng and Chan 2008). However, as already noted above, such studies have largely relied on a variable-centered approach to job insecurity. As a result, the way in which these objective characteristics may relate to different patterns of job insecurity is still not known. In the current study, we aimed to contribute to the existing literature by examining how a set of *objective characteristics* that reflect some occupational instability—temporary work, part-time contract, and the experience of organizational change—covary with the profiles of qualitative and quantitative job insecurity. Temporary or contingent work is one of the most frequently analyzed aspects of the job contract in job insecurity studies. Despite some findings showing that job insecurity may actually have stronger negative effects for permanent employees (e.g., Bernhard-Oettel et al. 2005; De Cuyper et al. 2010), it is quite unequivocally considered a condition increasing the likelihood of job insecurity (De Witte and Näswall 2003; Keim et al. 2014; Klandermans et al. 2010). Hence, we hypothesized that cumulated job insecurity profile(s) would be associated with temporary work. A similar implication may be drawn for organizational change experience, as it is usually seen as one of the main pre-conditions and causes for job insecurity to rise (Ashford et al. 1989; Keim et al. 2014). Subsequently, we took part-time versus full-time job into account. The evidence on the role of part-time employment in job insecurity perceptions is somewhat mixed (Bernhard-Oettel et al. 2005; De Witte et al. 2012; Näswall and De Witte 2003). However, as it may be indicative of an objectively weaker and thus more vulnerable position in the labor market, and objectively insecure employment status leads to job insecurity (e.g., Klandermans et al. 2010), we expected part-time workers to be more represented in the most “cumulated” job insecurity profile(s) compared to other profiles.

### Well-Being Outcomes

Furthermore, we aimed to investigate whether different job insecurity profiles show differential relationships with employee outcomes, as expressed in exhaustion, work engagement, and mental health. Drawing on Conservation of Resources Theory (Hobfoll 2001), these detrimental outcomes of job insecurity can be inferred due to so-called resource depletion processes. As a severe work stressor, job insecurity poses a threat to the valued status quo. It is then thought to trigger mental or behavioral attempts to restore it that may result in substantial loss of energy, withdrawal behaviors, and health impairment (e.g., Dekker and Schaufeli 1995; Vander Elst et al. 2014a and b). Furthermore, because quantitative job insecurity implies more loss (Greenhalgh and Rosenblatt 1984) and somewhat encompasses qualitative job insecurity (De Witte et al. 2015), it may be implied to have stronger detrimental outcomes. Unfortunately, this is only a theoretical implication at the moment, as existing literature comparing the effects of qualitative and quantitative dimensions of job insecurity is still scarce, and those few studies that have attempted to do so yield rather equivocal results. For instance, Hellgren et al. (1999) and Tu et al. (2019) found some support for the differential effects. In the first study, quantitative job insecurity was more strongly associated with employee well-being and the authors suggested that qualitative job insecurity might be primarily related to attitudinal outcomes. The second study reported a stronger relationship between quantitative job insecurity and stress symptoms, while qualitative job insecurity was related to lower work engagement. However, yet another comparative inquiry conducted by De Witte et al. (2010) did not detect major differences in terms of job insecurity outcomes and concluded that both job insecurity dimensions have similar negative effects. Adopting a person-centered approach, the current study may help to advance on this debate, as latent profile analyses allow for testing the potential cumulative effects. Even if both job insecurity dimensions may be expected to have similar outcomes, they have been shown to have separate negative effects controlling for each other. Hence, one may imply that the most “cumulated” profile (e.g., high on both job insecurity dimensions) should relate to particularly low health and well-being because the effects of the two job insecurity dimensions add up.

Based on the above, our second set of hypotheses is as follows:

*H2a*: Employees in vulnerable work situations (i.e., those with temporary contracts, part-time jobs, and organizational change experience) are more likely to fall into the most cumulated job insecurity profile;

*H2b:* Employee health and well-being (i.e., mental health and work engagement) are the lowest, whereas ill-being (i.e., exhaustion) is elevated in the most cumulated job insecurity profile.

## Subjects and Methods

### Procedure and Participants

The study was carried out in two separate samples of employees in Lithuania. The first sample consisted of 1077 employees representing both the public and the private sector (66% female, mean age = 37.12, *SD* = 12.51). The second sample, which was used as a cross-validation sample, consisted of 608 employees from public sector organizations (57% female, mean age = 41.86, *SD* = 10.99). A survey method was used to collect the data. It was held in Lithuanian. To this end, all scales were independently translated into the Lithuanian language by three experts. The best translation was then agreed upon by the study authors, which was ultimately double-checked by a linguist for accuracy and grammar. Participants completed the survey through an online platform. Both samples were drawn based on convenience sampling. Participants were contacted either through the HR management staff in their respective organizations or by directly spreading the invitation link to them via the mailing lists available to the authors. Irrespective of the recruitment method, all participants received identical information about the study. Before beginning the survey, they were introduced to the goal of the study and informed that the data would be treated confidentially and analyzed in an aggregate manner only. As the study was conducted online, the consent to participate was given by pressing the agree option that led the participant to the next page allowing to proceed with the survey. Participation in the study remained completely voluntary and participants could withdraw from it at any moment.

The research proposal for this study was approved by the Research Council of Lithuania. The study was conducted in compliance with the ethical guidelines and procedures of psychological research and following the code of ethics established by the Lithuanian Psychological Association. Since the study did not include clinical trials or research on vulnerable populations, it was not required to obtain a separate ethics approval for it.

### Measures

Background data were collected at the start of the survey. For sample 1, it included information about participants’ age, gender, experience of organizational change within the preceding year (i.e., no change, change related to one’s work, change unrelated to one’s work), type of contract (i.e., temporary or permanent), and work rate (i.e., full-time or part-time). Sample 2 participants came from organizations with recent change history, and since they represented the public sector with prevalent permanent full-time employment, only information about age and gender was collected in this sample.

#### Qualitative Job Insecurity

Qualitative job insecurity was measured using the scale developed by De Witte and De Cuyper, and further validated by Fischmann et al. (in press). This scale has been used in a number of studies over the recent years, such as Van den Broeck et al. (2014) and De Cuyper et al. (2019). It consists of four items measuring perceived negative changes in the overall job content and working conditions. A sample item: “I feel insecure about the characteristics and conditions of my job in the future”. Cronbach’s alphas were .86 in the first sample and .90 in the second sample.

#### Quantitative Job Insecurity

Quantitative job insecurity was measured with three items from the scale developed by De Witte (2000) and validated by Vander Elst et al. (2014 a and b). It reflects the perceived threat of losing the job itself. A sample item: “Chances are, I will soon lose my job”. Note that the original scale has four items, but one item had to be removed in the current study due to its high cross-loading on the qualitative job insecurity factor (see the Results section). Cronbach’s alphas were .86 in the first sample and .87 in the second sample. A five-point Likert type response format (from 1 – *completely disagree* to 5 *– completely agree*) was used for both the qualitative and quantitative job insecurity scales.

The below described measures were used in the second (i.e., cross-validation) sample only.

#### Work Engagement

Work engagement was measured with the nine-item version of the Utrecht Work Engagement Scale (UWES-9; Schaufeli and Bakker 2003). It measures such aspects of work engagement as vigor, absorption, and dedication. A sample item: “At my job, I feel strong and vigorous”. The answers were rated on a seven-point scale (from 0 – *never* to 6 – *always*). An overall score of work engagement was used in the present study. Cronbach’s alpha for the overall scale was .92.

#### Exhaustion

Exhaustion was measured with four negatively framed items from the Oldenburg Burnout Inventory (OLBI; Demerouti and Bakker 2008). A sample item: “After my work, I regularly feel worn out and weary”. The answers were rated on a five-point Likert type scale (from 1 – *completely disagree* to 5 – *completely agree*). Cronbach’s alpha was .87.

#### Mental Health

Mental health was measured with a five-item scale (MHI-5; Berwick et al. 1991), tapping into everyday affective states. The respondents were asked to indicate how frequently they felt in a certain way over the last six months. A sample item: “How much of the time during the last six months have you been a very nervous person?”. The answers were rated on a six-point scale (from 1 – *never* to 6 – *always*). Cronbach’s alpha was .90.

### Statistical Analyses

The analyses consisted of three stages. During the first stage, exploratory factor analyses (EFA) with Varimax rotation and confirmatory factor analyses (CFA) were conducted on job insecurity items. These analyses served two purposes. First, they allowed us to check whether qualitative and quantitative job insecurity are separate constructs. Second, we used job insecurity factor scores obtained during the EFA in further analyses. Given that qualitative and quantitative job insecurity are usually quite highly correlated and thus overlap to some extent, their factor scores served as better indicators for latent profile analysis. This initial step was carried out both on the first and on the second (i.e., cross-validation) sample. Subsequently, in the second stage, latent profile analyses (LPA; Lanza et al. 2003) were performed on both samples using MPlus version 7.11. As per guidelines in the literature (Nylund et al. 2007), we tested a two-profile solution first, increasing the number of profiles until the optimal solution was reached. Since there are no cut-off values for fit statistics in the LPA, the decision on the final profile solution was made based on a comparison of fit statistics for alternative profile models as well as on the interpretability of the profiles. The following fit statistics were inspected: the Akaike Information Criterion (AIC), the Bayesian Information Criterion (BIC), the sample-adjusted BIC (SaBIC), Lo-Mendell-Rubin likelihood ratio test (LMR), the Bootstrap Likelihood Ratio Test (BLRT), and entropy. Lower values of the AIC, BIC and SABIC indicate a better fitting model, whereas a non-significant value of the LMR and BLRT tests, obtained after comparing a k-profile model with a k-1 profile model, indicates that a more parsimonious model should be kept. Entropy informs about the classification accuracy, values closer to one indicating that a given profile solution fits better.

In the third stage, we tested the covariates of the final profile solution. Since the two samples did not contain exactly the same set of variables, we focused on the objective covariates in terms of occupational characteristics in sample 1, while sample 2 was used to analyze well-being and health outcomes. Specifically, using the data from sample 1, the expected distribution of occupational characteristics (i.e., type of contract, work rate, and type or experienced organizational change) was compared across the job insecurity profiles. To this end, the DCAT command in Mplus was used. It allows for examining categorical covariates without imposing bias to the LPA (Asparouhov and Muthén 2014). The cross-validation sample (i.e., sample 2) was then used for outcome comparison. Work engagement, exhaustion, and mental health were modeled as continuous distal outcomes using the BCH command (Asparouhov and Muthén 2018; Bakk and Vermunt 2016). It gives a comparison of the mean levels of each variable across the profiles and thus informs whether employees classified in a certain profile are more likely to score higher on any of these variables.

At this point, it may be important to note that since our analyses relied on a small number of latent profile indicators consisting of quantitative and qualitative job insecurity, a traditional “regression-based mindset” (cf. Zyphur 2009) might suggest an interaction analysis as an alternative to the LPA in predicting the well-being outcomes in this case. To illustrate and perhaps contrast the insights of each mindset, we have additionally conducted a series of tests where qualitative and quantitative job insecurity interact in predicting the outcomes.

## Results

### Descriptive Statistics

In the first sample (*N* = 1077), the mean level of qualitative job insecurity was 2.60 (*SD* = 0.81), whereas the mean level of quantitative job insecurity was 2.12 (*SD* = 0.77). With regard to the type of contract, 83.7% (*n* = 901) of participants were hired on a permanent basis, 11.8% (*n* = 127) held a temporary contract, and the remaining 4,5% *(n* = 49) indicated other option. Most participants worked full-time, 88.2% (*n* = 950), whereas the rest 11.8% (*n* = 127) had a part-time job. Concerning organizational change experience, 52.0% (*n* = 560) had not experienced any change, 18.3% (*n* = 197) had experienced change related to their work, 15.0% (*n* = 161) had experienced change that was unrelated to their work, and 14.7% (*n* = 159) did not know the answer. Correlations between job insecurity and the above-mentioned occupational characteristics are provided in Table 1. Furthermore, descriptive statistics for the variables in the cross-validation sample (*N* = 608) are provided in Table 2 and inform about the means, standard deviations, inter-correlations between these variables.

**Table 1:** Correlations between job insecurity, background variables, and occupational characteristics in the initial sample (*N* = 1077)

Variables	1	2	3	4	5	6	7
1. Qualitative JI	–						
2. Quantitative JI	.54^***^	–					
3. Age	.13^***^	.21^***^	–				
4. Gender (male)	−.04	<.01	−.03	–			
5. Contract (permanent)	−.01	−.07^*^	.17^***^	.03	–		
6. Work rate (full-time)	−.01	−.07^*^	.14^***^	.04	.09^**^	–	
7. Change-related	.16^***^	.04	.16^***^	−.06	−.02	.11^**^	–
8. Change-unrelated	−.05	<.01	.09^*^	−.07	.04	<.01	n/a

***Notes.*** JI = job insecurity. Change-related = organizational change related to one’s work. Change-unrelated = organizational change unrelated to one’s work. Reference category for contract is temporary contract. Reference category for work rate is part-time. Reference category for both organizational change variables is no change. Change-related and change-unrelated could not be correlated because they are exclusive of each other (the participants had to mark only one option). ^***^p < .001, ^**^p < .01, ^*^p < .05

**Table 2:** Variable means and inter-correlations in the cross-validation sample (*N* = 608)

	*M*	*SD*	1	2	3	4
1. Quantitative JI	2.16	0.79				
2. Qualitative JI	2.66	0.88	.61^***^			
3. Work engagement	4.14	1.10	−.22^***^	−.32^***^		
4. Exhaustion	2.73	0.82	.30^***^	.38^***^	−.34^***^	
5. Mental health	4.30	0.87	−.35^***^	−.46^***^	.44^***^	−.74^***^

*Note*. JI = job insecurity. Exhaustion was tested on a reduced sample of 491 employees

^***^p < .001

### Factor Analyses

The results from EFA showed a clear two-factor solution representing the qualitative and quantitative job insecurity dimensions. However, one quantitative job insecurity item had to be removed from the scale due to its high cross-loading on both factors, the same results being replicated on both samples. After removing it, the EFA results from the first sample (*N* = 1077) showed a two-factor structure that explained 74% of variance, with factor loadings from .69 to .88. A similar output was obtained in the cross-validation sample (*N* = 608), where a two-factor solution explained 79% of variance, with factor loadings ranging from .78 to .90.

The CFA, which was additionally conducted to check for construct validity, showed good fit indices for the two-factor model in the first sample, χ^2^(12) = 66.40, *p* < .001, TLI = .978, CFI = .988, RMSEA = .065, and they were similar in the second sample, χ^2^(12) = 70.11, *p* < .001, TLI = .967, CFI = .981, RMSEA = .089. The χ^2^ difference statistics supported the two-factor model over the one-factor model in both cases, with Δχ^2^(1) = 687.21, *p* < .001 in the first sample and Δχ^2^(1) = 526.93, *p* < .001 in the second sample.

### Latent Profile Analyses

A comparison of alternative profile solutions in both samples is summarized in Table 3. The results did not provide a clear answer as to which profile solution was the best. In sample 1, a three-profile solution yielded well-interpretable profiles with adequate fit indices with the single exception of the LMR test that was non-significant. In comparison, a rather steep drop in entropy, a non-significant LMR test, and at least one very small profile were observed for the four-profile solution, whereas the five-profile model had similar characteristics despite an increase in entropy. Hence, based on the summary of fit statistics and on the interpretability of the profiles, the three-profile solution was chosen as the optimal one in the first sample.

**Table 3:** Latent profile solutions and their fit statistics

	AIC	BIC	SABIC	LMR (*p*)	BLRT (*p*)	Entropy	Smallest profile (%)
*Sample 1*							
2-profile solution	6060.355	6095.229	6072.996	.002	<.001	0.908	4.3
3-profile solution	6034.316	6084.135	6052.373	.101	<.001	0.832	4.9
4-profile solution	5999.776	6064.541	6023.250	.165	<.001	0.647	1.6
5-profile solution	5951.945	6031.656	5980.837	.297	<.001	0.795	2.5
*Sample 2*							
2-profile solution	3424.789	3455.661	3433.437	.001	<.001	0.935	3.6
3-profile solution	3411.996	3456.098	3424.350	.003	<.001	0.896	3.6
4-profile solution	3397.643	3454.975	3413.703	.019	<.001	0.676	3.2
5-profile solution	3365.738	3436.301	3385.505	.296	<.001	0.818	3.5

*Note*. Sample 1 = initial sample (*N* = 1077). Sample 2 = cross-validation sample (*N* = 608)

**Fig. 1:**
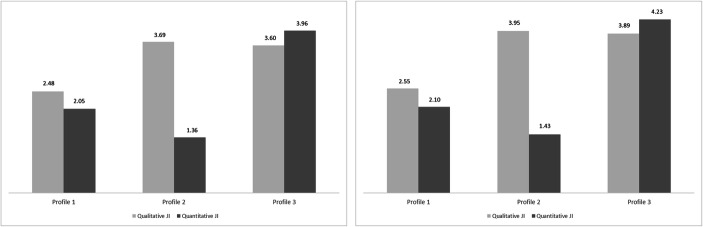
a. Job insecurity profiles in the initial sample. Figure based on scale scores. b. Job insecurity profiles in the cross-validation sample. Figure based on scale scores.

In sample 2, a three-profile solution also fitted to the data well regarding all fit statistics. In comparison, a four-profile solution showed a drop in entropy, whereas a five-profile solution had a non-significant LMR test and added little interpretability compared to the three-profile model. For this reason, we consider that the results of sample 1 were replicated in sample 2, yielding the three-profile solution as the optimal one. A graphical representation of the profiles is provided in Figs. 1a and 1b and shows a very similar results pattern across the two samples (see Appendix 1 for additional similarity analyses following the guidelines in Morin et al. 2016). Please note that while the analysis was based on factor scores, the figures show scale scores to increase the interpretability of the results. We labelled profile 1 as the balanced low job insecurity profile (89.2% in sample 1 and 92.5% in sample 2), profile 2 was labelled as the qualitative job insecurity dominant profile (4.9% and 3.6%, respectively), whereas profile 3 was labelled as balanced high job insecurity profile (5.9% and 3.9%, respectively).

### Covariate Analyses

Categorical covariate analyses conducted on sample 1 showed several significant pairwise profile differences. Specifically, the balanced high job insecurity profile significantly differed from the balanced low job insecurity profile in terms of the distribution of employees with temporary versus permanent type of contract, χ^2^(1) = 5.10, *p* = .024. The balanced high job insecurity profile compared with the balanced low job insecurity profile was associated with a higher probability of having a temporary contract. Moreover, the distribution of employees with temporary versus permanent work contract was different across the balanced high job insecurity profile and the qualitative job insecurity dominant profile, χ^2^(1) = 4.27, *p* = .039. The balanced high job insecurity profile compared with the qualitative job insecurity dominant profile was associated with higher probability of having a temporary contract. In terms of work rate, a significant difference was also observed between the balanced high job insecurity profile and the qualitative job insecurity dominant profile, χ^2^(1) = 4.35, *p* = .037. The balanced high job insecurity profile was associated with a higher probability of having a part-time job. Subsequently, there was a significant difference between the balanced low job insecurity profile and the qualitative job insecurity dominant profile in terms of participants’ organizational change experience, χ^2^(2) = 6.77, *p* = .034. The balanced low job insecurity profile, compared to the qualitative job insecurity dominant profile, was associated with a lower probability of having experienced work-related organizational change and a higher probability of having experienced organizational change that was unrelated to one’s work. Probability estimates within the profiles are summarized in Table 4. The overall between-profile test, however, revealed non-significant results in the expected distribution of temporary employment (χ^2^(2) = 5.34, *p* = .069), part-time contract (χ^2^(2) = 4.52, *p* = .104), or organizational change experience (χ^2^(4) = 7.12, *p* = .130), which may be due to quite different size and variability of the compared profiles.

**Table 4:** Occupational characteristics probability estimates across the job insecurity profiles

	Profile 1	Profile 2	Profile 3	
	Probability estimates			Profile differences
Type of contract:				1 vs 3, 2 vs 3
Temporary	.11	.08	.31	
Permanent	.89	.92	.69	
Work rate:				2 vs 3
Full-time	.89	.97	.76	
Part-time	.11	.03	.24	
Change:				1 vs 2
No change	.65	.52	.59	
Work-related	.15	.36	.25	
Work-unrelated	.20	.12	.16	

*Note*. Profile 1 = balanced low job insecurity. Profile 2 = qualitative job insecurity dominant

Profile 3 = balanced high job insecurity. Profile differences column informs about significant

pairwise differences between the profiles with regard to the distribution of occupational characteristics

Finally, covariate analyses based on the BCH procedure on sample 2 revealed several differences across the job insecurity profiles in terms of the investigated distal well-being outcomes. Specifically, overall between-profile comparisons showed significant differences in the mean values of work engagement, χ^2^(2) = 10.65, *p* = .005 and mental health, χ^2^(2) = 12.04, *p* = .002. No significant between-profile differences were found in the mean values of exhaustion, χ^2^(2) = 3.92, *p* = .141. Pairwise comparisons are provided in Table 5 and show that mental health self-ratings were most positive in the balanced low job insecurity profile and they differed in a significant manner from those in the balanced high job insecurity profile and the qualitative job insecurity dominant profile. A similar pattern was observed with work engagement as a distal outcome. The mean values of work engagement were the highest in the balanced low job insecurity profile and they significantly differed from those in the two remaining profiles.

**Table 5:** Mean scores of employee health and well-being outcomes across the job insecurity profiles

	Job insecurity profile		
Outcome variables	Balanced low	Qualitative JI dominant	Balanced high
Work engagement	4.22^a,b^	2.83^b^	3.61^a^
Exhaustion	2.73	2.55	3.19
Mental health	4.34^a,b^	3.82^b^	3.62^a^

*Note*. Profiles sharing the same superscript letter differ between each other in the mean values of a given outcome variable (*p* < .05). A higher mental health score indicates better mental health. JI = job insecurity

### Contrasting LPA and Regression-Based Findings

To complete the results with insights from both analytic mindsets, the regression-based findings are additionally reported in Appendix 2. For comparison, we may see that qualitative and quantitative job insecurity only interacted in predicting work engagement but not regarding other outcomes. Similar to the LPA results, work engagement was the highest under the low qualitative-low quantitative job insecurity condition and the lowest when qualitative job insecurity was high. However, one should keep in mind that not all combinations depicted in the interaction analysis turned out to be equally realistic. One should be especially careful interpreting the low qualitative-high quantitative job insecurity condition because we know from the LPA that such combination barely exists in our sample (hence, it likely relies on a very small number of subjects). Moreover, no significant interaction was found for mental health as the outcome, whereas a person-centered approach is suggestive of significant differences between the profiles with regard to it.

## Discussion

This study aimed to identify the latent profiles of qualitative and quantitative job insecurity and link them to participants’ work situation as well as to health and well-being outcomes. This represents a novel way of investigating job insecurity and generates meaningful new knowledge as we unravel and cross-validate the different patterns in which the two dimensions of job insecurity naturally co-occur. These patterns or profiles may be thought of as a repertoire of increasingly precarious situations that employees can encounter. In today’s turbulent world of work, such knowledge is particularly useful because it provides a glimpse into how anticipated changes and losses at work (be it the loss of a valued feature of the job or the job itself) translate into vulnerability experiences that are then linked to various objective preconditions and subjective detrimental outcomes.

In the current study, we hypothesized that qualitative and quantitative job insecurity dimensions would add up and would be particularly likely to manifest in vulnerable, less stable occupational situations (e.g., characterized by temporary, part-time or change-encompassing jobs). In addition, we implied that they might produce more negative effects than any single qualitative job insecurity dimension. In this way, our study enriches the theoretical understanding of job insecurity by testing whether the occurrence of its qualitative and quantitative dimensions is mutually inclusive (i.e., whether for a given person experiencing high levels of one aspect of job insecurity would at the same time imply experiencing elevated levels of the other one). Given only scarce attempts to investigate how these two job insecurity dimensions combine to produce detrimental effects (e.g., Chirumbolo et al. 2017; De Cuyper et al. 2019), the present study significantly contributes to existing literature. Specifically, we advance on Chirumbolo et al. (2017) by testing the relative co-occurrence of qualitative and quantitative job insecurity in the form of latent profiles, which provides a more elaborate and fine-grained picture on how they coincide. We also add on previous latent profile analyses by De Cuyper et al. (2019) and their career-focused findings by showing how the observed job insecurity profiles relate to a completely different set of health and well-being outcomes.

In line with Hypothesis 1, our first notable finding was that high levels of qualitative job insecurity indeed tended to occur on their own, while those of quantitative job insecurity did not. Interestingly, our results only partially overlap with those of De Cuyper et al. (2019), who identified five job insecurity profiles that ranged from relatively secure to very insecure, three profiles being dominated by qualitative job insecurity in terms of absolute scale scores. For comparison, in our study, three salient profiles emerged, also ranging from very secure to very insecure, with just one overall qualitative job insecurity dominant profile. Given that any classification is contingent on the sample, it may have been the reason of such discrepancy. De Cuyper et al. (2019) used a larger-sized sample, which presumably offers more power to detect profiles with quite subtle differences between them. Moreover, they estimated the profiles based on correlated job insecurity factor scores, whereas in the current study we aimed to circumvent this issue by using the scores obtained from an orthogonal rotation, which helps to elucidate the differences between qualitative and quantitative job insecurity. A major contribution of our current findings is that they address how the two job insecurity dimensions are intrinsically interwoven, which has not received much attention before. We offer practically relevant evidence about their relative co-occurrence, suggesting that the experience of quantitative job insecurity may be cumulative: It seems to be indispensably accompanied by qualitative job insecurity, whereas the qualitative dimension of job insecurity did occur independently. The size of the qualitative job insecurity dominant and the balanced high job insecurity profiles was rather small in both samples, the balanced low job insecurity profile representing the majority of the study participants. However, we believe that such results correspond to the reality quite well. Both qualitative job insecurity dominant and balanced high job insecurity profiles denote extremities in job insecurity that naturally are observed in a small proportion of the population (De Cuyper et al. 2019; Eurofound 2017). Somewhat similar findings were also observed by Kinnunen et al. (2014), who used growth mixture modeling and found that extreme profiles (i.e., denoted by increasing job insecurity) represented less than 10% of the sample.

A novel insight offered by our findings is that these extremities may qualitatively differ in their shape, which leads to different implications for employees. Some sources in the job insecurity literature maintain that quantitative job insecurity is a more severe stressor (De Witte 1999; De Witte et al. 2010; Greenhalgh and Rosenblatt 1984). Moreover, Chirumbolo et al.’ (2017) study implies that the negative effects of quantitative job insecurity are explained by qualitative job insecurity. The reasoning based on our current findings goes beyond that, offering an explanation for why this may be the case. Specifically, our study hypothesized that quantitative job insecurity might encompass the negative aspects of qualitative job insecurity, resulting in a cumulated profile that is related to the most vulnerable occupational situations. The results of covariate analyses partly supported this claim, corroborating Hypothesis 2a. First, the findings of the current study showed evidence that compared to the qualitative job insecurity dominant profile, the most extreme (i.e., balanced high job insecurity) profile was characterized by more vulnerable employees in terms of temporary contracts and part-time jobs. This is in line with the literature suggesting that objective occupational characteristics contribute to an increased likelihood of encountering job insecurity (see De Witte and Näswall 2003; Keim et al. 2014; Kinnunen and Nätti 1994; Shoss 2017). Moreover, our findings additionally draw attention to the fact that certain groups of employees, such as those in contingent jobs, may be particularly at risk of experiencing cumulative forms of job insecurity.

Distal outcome analysis, however, did not support the claim (i.e., Hypothesis 2b) that the extreme high job insecurity profile would result in cumulative negative effects. According to the mean level comparisons, both qualitative job insecurity dominant and balanced high job insecurity profiles were associated with lower work engagement and mental health ratings than the balanced low job insecurity profile. Despite the lowest mean values of mental health and the highest ratings of exhaustion, the balanced high job insecurity profile did not show to be significantly different from the qualitative job insecurity dominant profile in terms of employee health and well-being. For comparison, except for work engagement, supplementary variable-centered regression analyses did not reveal interaction effects in predicting the outcomes, showing that the effects of qualitative job insecurity were only marginally determined but not strengthened by the levels of quantitative job insecurity. These results, however, point to the relative importance of single dimensions placing more emphasis on qualitative job insecurity. Hence, as previously noted by Gabriel et al. (2015), a person-centered approach offers a unique complementary insight to regression-based analyses. In our case, it may help clarify why qualitative job insecurity appears as a more salient predictor. Specifically, one explanation could be linked to the observation that based on latent profiles employees in the current study were much more likely to encounter qualitative job insecurity without its quantitative counterpart, but not the other way around. Thus, qualitative job insecurity seems to be present in the vast majority of job-insecure situations. Such findings also add to the discussion in the existing literature as to which dimension of job insecurity – qualitative or (cumulated) quantitative – may produce more severe detrimental effects (e.g., De Witte et al. 2010; Tu et al. 2019). Our findings suggest that qualitative job insecurity alone may be severe enough for the detrimental outcomes to occur. This implication is particularly relevant in times of turbulent economies (e.g., due to the recent COVID-19 crisis), when many companies are forced to implement changes in their size, structure, or the ways of working. The risk of losing one’s job (i.e., quantitative job insecurity) would be considered a well-recognized and “legitimate” threat to employee well-being in such cases. Whereas qualitative job insecurity is easy to overlook because it has the label of being somewhat milder and inevitable. In light of this, our findings are alerting as they show that the latter form of job insecurity can be substantially detrimental, even if it is not accompanied by the risk of losing the job. At this point, however, we cannot explain why the strength of the detrimental outcomes did not increase in the balanced high job insecurity profile. Perhaps the non-significant difference between the balanced high job insecurity profile and the qualitative job insecurity dominant profile could be due to the very small size of these profiles. For this reason, true mean score differences in the outcomes may be more difficult to detect. Moreover, the cross-validation sample used for distal outcome analyses consisted of public sector employees only, who had encountered some form on organizational change. It is possible that qualitative job insecurity was a more pertinent job insecurity dimension for them, and this may have somewhat affected the results.

Notably, although the current study did not provide a straightforward answer about the potential cumulative effects of quantitative and qualitative job insecurity, this does not minimize the importance of it as a stressor because the mere presence of the qualitative dimension of job insecurity may already signal a vulnerable situation. Since our findings showed that qualitatively job insecurity dominant profile was also related to the experience of organizational change, this indirectly suggests that, if not properly addressed, any type of anticipated involuntary work-related change may represent a risk of impaired health and well-being among employees. It is an important message to managers and human resources professionals emphasizing the need for careful and transparent planning of organizational change initiatives (see De Witte et al. 2015 for more recommendations).

### Limitations and Future Research Implications

The current study bears some limitations that must be taken into account when interpreting the results. First, any analysis that classifies people into categories or profiles is bound to the sample used for the study. While the strong point of the current study is that the initial profile solution was cross-validated on a different sample, sample composition and characteristics may still have affected the results. This is particularly true for the cross-validation sample that was homogeneous, consisting of public sector employees only. Hence, future replications based on larger and more heterogeneous samples would be sensible in order to address the potential cumulative effects question in a more precise manner.

Second, because the current study was based on the conceptualization of qualitative and quantitative job insecurity, the latent profiles analyses were based on two indicators only, which is quite restrictive. While this was the only available option in our study, further studies may consider assessing separate aspects of qualitative job insecurity (such as salary, growth opportunities, etc.) and then use them as indicators in LPA.

Furthermore, it is important to note that job insecurity is naturally not equally distributed in the working population in terms of its severity, usually a rather small proportion of the sample reporting to be extremely insecure (e.g., Eurofound 2017). This apparently results in a large profile of low to moderate job insecurity versus one or more small profiles characterized by high job insecurity. While perhaps such a tendency reflects the actual situation in the labor market, it encumbers profile comparisons in terms of their distal outcomes. Finally, it has to be noted that this study included only a small set of employee health and well-being outcomes. Including a wider scope of outcome variables in the future studies based on person-centered analyses is essential, for it may help to identify whether any differential or cumulative effects exist between different job insecurity profiles.

## Conclusion

Three qualitative and quantitative job insecurity latent profiles were observed: the balanced low job insecurity profile, the balanced high job insecurity profile, and the qualitative job insecurity dominant profile. This demonstrates that while quantitative job insecurity comes along with qualitative job insecurity, the latter may occur on its own. As hypothesized, temporary and part-time employees had a higher probability of falling into the balanced high job insecurity profile. Moreover, the qualitative job insecurity dominant profile, but not the balanced high job insecurity profile, differed from the low job insecurity profile in terms of organizational change experience. Somewhat unexpectedly, the balanced high and the qualitative job insecurity dominant profiles did not differ in terms of health and well-being outcomes: both were related to significantly lower mean levels of mental health and work engagement compared with the low job insecurity profile. This is an interesting result suggesting that qualitative job insecurity alone may be powerful enough to bring detrimental outcomes. This does not support, however, our initial claim about the cumulative effects of qualitative and quantitative job insecurity, which asks for more research in this direction.

## Supplementary Information

ESM 1 (DOCX 14 kb)

ESM 2 (DOCX 56 kb)

## Footnotes

1 For an alternative conceptualization of affective and cognitive job insecurity, see Jiang and Lavaysse (2018)
