# Supplementary material for: Deconstructing Job Insecurity: Do its Qualitative and Quantitative Dimensions Add Up?
Source: Occup Health Sci. 2021 Aug 12;5(3):415–35. doi: 10.1007/s41542-021-00096-3 (PMC8359915; doi:10.1007/s41542-021-00096-3)
Supplement: Supplementary file 1 — (DOCX 14 kb) [file 41542_2021_96_MOESM1_ESM.docx]

**Appendix A**

**Similarity of the 3-profile solution in Sample 1 and Sample 2**

Latent profile solution similarity tests across the two samples were carried out following the procedure suggested by Morin et al. (2016). The results showed the latent profiles to be comparable in Sample 1 and Sample 2 regarding four types of similarity reported in the table: configural, structural, dispersion, distributional.

**Table A1.** Similarity test results

| Type of similarity | AIC | BIC | SABIC | Entropy |
| --- | --- | --- | --- | --- |
| Configural | 11651.937 | 11765.957 | 11699.243 | 0.911 |
| Structural | 11641.082 | 11722.525 | 11674.872 | 0.918 |
| Dispersion | 11637.085 | 11707.669 | 11666.369 | 0.918 |
| Distributional | 11633.155 | 11692.879 | 11657.934 | 0.919 |

*Notes.* Configural similarity refers to the number of extracted profiles in the compared samples. Structural similarity refers to within-profile mean level similarity across the compared samples. Dispersion similarity refers to similarity in within-profile variability. Distributional similarity tests compare the size of the profiles in a given profile solution across the two samples. Similarity tests are based on model comparison, where the configural model is taken as a baseline and incremental constraints are added to it. Lower values of information criteria in a more constrained model indicate that its fit has not decreased due to the added constraints.
