# Supplementary material for: Deconstructing Job Insecurity: Do its Qualitative and Quantitative Dimensions Add Up?
Source: Occup Health Sci. 2021 Aug 12;5(3):415–35. doi: 10.1007/s41542-021-00096-3 (PMC8359915; doi:10.1007/s41542-021-00096-3)
Supplement: Supplementary file 2 — (DOCX 56 kb) [file 41542_2021_96_MOESM2_ESM.docx]

**Appendix B**

**Interaction of qualitative and quantitative job insecurity in predicting employee**

**health and well-being outcomes**

**Table B1**. Interaction analyses results

|  | Outcome variables | | |
| --- | --- | --- | --- |
| Predictors | Work engagement | Exhaustion | Mental health |
| Qualitative JI | -.31*** | .27*** | -.35*** |
| Quantitative JI | -.16*** | .16*** | -.20*** |
| JI interaction | .08* | .01 | <.001 |
| R^2^ main effects model | .10 | .15 | .22 |
| R^2^ change due to interaction | .01* | <.001 | <.001 |

*Notes*. JI = job insecurity. JI interaction = interaction between qualitative and quantitative job insecurity. Analyses are based on job insecurity factor scores, as they were also used as profile indicators in the LPA. **p* < .05, ****p* < .001.


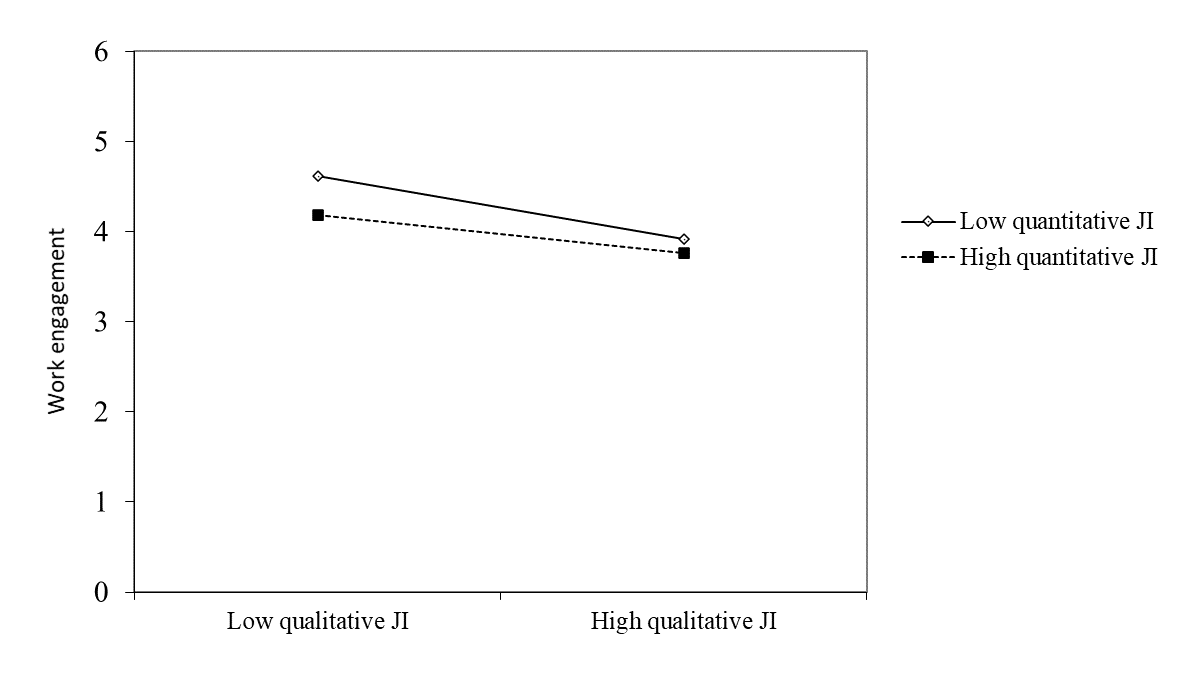


**Figure B1.** Significant interaction between qualitative and quantitative job insecurity in predicting work engagement.
